# Use of the ACTH challenge test to identify the predominant glucocorticoid in the southern sea otter (*Enhydra lutris nereis*)

**DOI:** 10.1093/conphys/coz116

**Published:** 2020-01-24

**Authors:** M J Murray, M A Young, R M Santymire

**Affiliations:** 1 Monterey Bay Aquarium, 886 Cannery Row, Monterey, CA 93940, USA; 2 Conservation & Science Department, Lincoln Park Zoo, 2001 N. Clark St., Chicago, IL 60614, USA

**Keywords:** corticosterone, cortisol, enzyme immunoassay, physiological validation, stress physiology

## Abstract

After nearly being hunted to extinction during the fur trade of the late 20th Century, sea otter (*Enhydra lutris*) populations have recovered to varying degrees of their historical range. While overall population numbers and range have increased, there are regions in which expansion has occurred at a slower rate and/or animal numbers have decreased, which may be a result of chronic stress from a variety of sources. Some have employed glucocorticoid analysis in their attempts to validate these explanations. Our goal was to conduct a controlled study using sea otters managed under human care to validate the use of serum glucocorticoid analysis to monitor stress physiology in the sea otter. We used a standard ACTH challenge test to compare cortisol and corticosterone responses, thereby identifying the primary glucocorticoid in the sea otter. Fourteen sea otters of both sexes (five males, nine females), including juveniles, sub-adults and adults, participated in the study. The results of the testing supported cortisol as the primary glucocorticoid in the sea otter. Sex and age did not affect how the individual responded to the ACTH or saline injection. Interestingly, the saline injection not only confirmed the effects of the ACTH on glucocorticoid release from the adrenal glands but also provided information on how long it takes the sea otter’s glucocorticoid levels to return to baseline after capture and sedation. The insight gained from this study will aid in future efforts to better understand the role of stress in free-ranging sea otter populations. Recognition of the primary glucocorticoid will facilitate evaluation of more stable biological material, such as fur or whiskers, which tend to be less affected by the diurnal cycling of glucocorticoids.

## Introduction

Environmental changes driven by anthropogenic activities, such as pollution and climate change, are affecting the health and success of wildlife populations (Machalaba *et al*., 2018; Noyes *et al*., 2009; Nuijten *et al*., 2016; Swarup and Patra, 2005; Van Hemert *et al*., 2014). These pressures can create a more stochastic environment that may result to increase stress for wildlife that are trying to cope with this variability. This elevated stress may lead to a more vulnerable and susceptible population as evident by the increased rate of emerging infectious diseases (Harper and Austad, 2004). For example, the protozoan parasite, *Toxoplasma gondii*, a typical pathogen of terrestrial animals including humans, was found in 42% live and 62% dead southern sea otter (*Enhydra lutris nereis*) sampled between 1997 and 2001 (Miller *et al*., 2002). Because it lives in nearshore habitats along the California coast from Point Conception to Half Moon Bay, the threatened southern sea otter may be particularly vulnerable to anthropogenic pressures, such as pollution from freshwater runoff which was the major risk factor for *T. gondii* exposure (Miller *et al*., 2002).

Prior to near extirpation by the fur trade, sea otters were apex predators in nearshore ecosystems extending from the Pacific coast of the Baja Peninsula north and west to the northern islands of Japan (Riedman *et al*., 1990). Once large-scale hunting was discontinued, sea otter populations grew through a combination of natural range expansion and re-introduction (Riedman *et al*., 1990). The rate of population growth is limited by varying combinations of geography and bathymetry; resource, especially food, availability; predation; disease; and loss of genetic diversity (Estes, 1990; Tinker *et al*., 2017). The southern sea otter’s recovery has been sluggish due to a number of concurrent factors as described above, as well as the significant population-level effects of non-consumptive shark predation (Tinker *et al*., 2016). The genetic bottleneck, which was a result of the fur trade, may also be impacting population recovery (Bodkin *et al*., 1999; Gagne *et al*., 2018; Larson *et al*., 2002a, b; Larson *et al*., 2012). It has been postulated that loss of genetic diversity may cause chronic stress in individual sea otters (Larson *et al*., 2009).

Surprisingly, standard serum-based endocrinological methods have not been used to identify the primary glucocorticoid in sea otters. Prior studies comparing glucocorticoids (GC) in sea otters identified inter-population differences in corticosterone and concluded that corticosterone is the primary stress hormone in sea otters (Larson *et al*., 2009). However, the validity of these studies may be questioned without prior understanding of the nature of sea otter adrenocortical response to stressors.

One approach to evaluate the physiological impact of a spectrum of stressors on the health of individuals or populations is to monitor GCs released from the adrenal cortex when the hypothalamic–pituitary–adrenal (HPA) axis is activated in response to a stressor (Reeder and Kramer, 2005). These hormones help an individual cope with a stressor in part by providing energy through increased gluconeogenesis, decreased glucose use and decreased cellular sensitivity to insulin. GCs can reduce inflammation by reducing cytokine production and suppressing white blood cells and several interleukins (ILs) including IL-1 and IL-2 (Welsh *et al*. 1999). Chronic exposure to endogenous GCs can have a negative effect due to hepatocellular degeneration, loss of body condition as a result of muscle wasting, neuronal cell malfunction, behavioural and cognitive anomalies and immunosuppression making the individual susceptible to a suite of primary and opportunistic pathogens (Boonstra, 2005; Wingfield, 2005; Wingfield and Romero, 2001).

**Figure 1:**
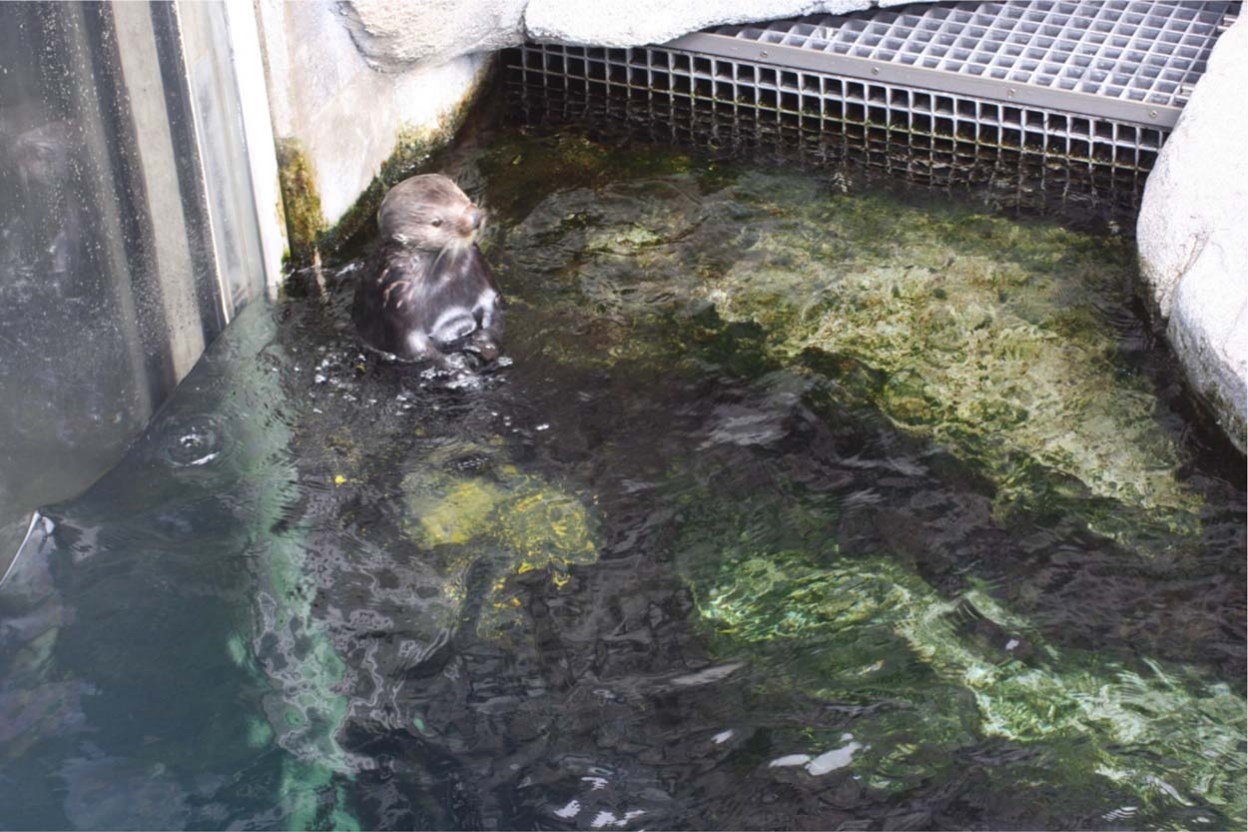
Appearance of faeces from an aquarium resident sea otter immediately after being voided in the water column. Photo taken by Dr M. Murray

Typically, blood samples are avoided when monitoring the stress physiology of non-domestic species, because GCs can increase within minutes after capture or restraint in mammals and birds (Harper and Austad, 2000; Millspaugh and Washburn, 2004; Mostl and Palme, 2002; Touma and Palme, 2005). Hormone concentrations in the blood may not be an accurate representation of overall hormonal activity, but only reflect hormone levels at a particular point of time. Additionally, there is evidence that most species have some degree of diurnal variability in circulating levels of GCs making interpretation of solitary blood levels problematic (Heintz *et al*., 2011; Kolevska *et al*., 2003). For this reason, non-invasive methods, such as faecal or urine hormone metabolite analysis, are used for many species, including sea otters (Wasser *et al*., 2000). Unfortunately, faecal and urine collection can be difficult in some species, such as aquatic animals like the sea otter particularly when their diets are free of non-digestible shell and chiton because the faeces are not well formed and can disperse quickly in water (Fig. 1). In these cases, hormones obtained from blood may be the best option for gathering information about an animal’s stress physiology. If this is the preferred methodology, proper analysis is needed to ensure that capture and anaesthesia methods are not resulting in elevated GCs.

To ensure that the hormone concentration is an accurate representation of the animal’s physiology, hormone analysis should be validated. In the case of GC analysis, validation can be accomplished by using adrenocorticotropic hormone (ACTH) to stimulate adrenocortical activity (Touma and Palme, 2005; Wasser *et al*., 2000). ACTH is a component of the HPA axis. After release from the anterior pituitary gland, it stimulates the adrenal cortex to release GCs. Therefore, ACTH can be administered by injection; then biological samples, such as blood, urine, faeces, are collected and GCs, like cortisol and corticosterone, can be measured (Touma and Palme, 2005).

The goal of this study was to evaluate the stress physiology of southern sea otters utilizing standard ACTH challenge testing in an effort to identify the dominant GC, cortisol or corticosterone in serum. Wasser and co-authors (2000) found that cortisol was the more appropriate GC after an ACTH challenge in a male Alaskan sea otter (*Enhydra lutris kenyoni*). Identification of the sea otter’s primary serum stress hormone may provide opportunities to use other samples too, including saliva or fur to evaluate stress. GC levels in hair, in particular, may be a good biomarker for long-term stress, as it has been demonstrated to be more refractory to normal circadian cycles in other species (Gow *et al.*, 2010; MacBeth *et al.*, 2010; Russell *et al.*, 2012; Sheriff *et al.*, 2011). In addition to the primary study goal of identifying the primary glucocorticoid, the study’s design has provided insight into the effect of sedation, age and sex on HPA response.

## Materials and methods

### Study animals

This study was performed under the auspices of US Fish & Wildlife Service Threatened Marine Mammal Scientific Research permit #MA-186914-2 and prior review and approval by the IACUCs of the Monterey Bay Aquarium (protocol #14-01) and the Lincoln Park Zoo (protocol #2014-028).

Sea otters used for challenge testing were either animals permanently housed under human care at the Monterey Bay Aquarium (*n* = 5; sea otter #479, 567, 649, 654 and 5059) or post-weaned surrogate reared pups approaching release age at the Monterey Bay Aquarium (*n* = 9; sea otter #595, 623, 657, 671, 673, 684, 685, 687 and 696). All study animals were determined to be clinically healthy based on physical examinations and CBC/serum chemistry evaluations prior to initiation of the testing. Ages varied from 7 months to 14 years of age, and the otters had been on site at the Aquarium or under human care for at least 6 months. All were considered to be well adapted to their living conditions.

For the purposes of this study, each otter served as its own control. Otters were handled twice for the evaluation, the first being the control response to an injection of sterile physiological saline, 0.9% NaCl, (Physiological Saline Solution, Nova-Tech, Inc., Grand Island, NE, USA) followed at least 72 h later by an ACTH challenge. We elected to perform the control challenges first, as we were confident that there would be no significant response or side effect associated with a solitary dose of physiological saline. However, this degree of certainty was not present for administration of ACTH, and we were concerned about a protracted effect on the HPA axis. This could have affected the saline trial if it was conducted after the ACTH trial. Animals were handled, treated and sampled in the same manner each time. Following an overnight fasting, the sea otter was removed from its holding tank with a dip net, immediately physically restrained and hand-injected with standard immobilization doses of fentanyl citrate, 0.22 mg/kg (fentanyl citrate injection, Central Avenue Pharmacy, Pacific Grove, CA, USA) and midazolam hydrochloride, 0.07 mg/kg (midazolam HCl Injection, Alvogen, Inc., Pine Brook, NJ, USA) intramuscularly (Monson *et al*., 2001) all within 5 min. Otters were then transferred into a cool, dark, quiet box undisturbed for 8–10 min to allow the sedatives to take effect. Following the procedure, the effects of fentanyl citrate were reversed with an intramuscular injection of naltrexone hydrochloride, 1.1 mg/kg (naltrexone HCl injection, ZooPharm, Ft. Collins, CO, USA). No untoward effects associated with the immobilization were noted in any of the study animals.

Upon removal from the sedation box, a blood sample was collected from the jugular vein (pre-injection). An intramuscular injection of 1.0 ml physiological saline or 40 IU ACTH gel (ACTH gel; Injection, Roadrunner Pharmacy, Phoenix, AZ, USA) was administered within 30 s of pre-injection. Subsequent sampling occurred 15 and 30 min post saline/ACTH injection. Blood samples were collected into plain glass blood collection tubes (Monoject Blood Collection Tube, Covidien, LLC, Mansfield, MA, USA) allowed to sit undisturbed for 15 min to allow a clot to form and then centrifuged at 3500 rpm. Serum was harvested, aliquoted to 0.5-ml volumes and frozen at −80°F. Samples, both control and ACTH challenge, were shipped via overnight express on dry ice to Davee Center for Epidemiology and Endocrinology laboratory at Lincoln Park Zoo (Chicago, IL) for analysis.

### Glucocorticoid analysis

GC concentrations were analyzed for both cortisol and corticosterone enzyme immunoassays (EIA) using previously described methods (Munro and Stabenfeldt, 1984; Santymire and Armstrong, 2010). The corticosterone EIA polyclonal antiserum (CJM006; C. Munro, University of California, Davis, CA, USA) and the horseradish peroxidase (HRP) ligand was diluted to 1: 6000 and 1:20 000, respectively. Antiserum cross-reactivities for corticosterone were corticosterone, 100%; desoxycorticosterone, 14.25%; tetrahydrocorticosterone, 0.9%; 11-deoxycortisol, 0.03%; prednisone, < 0.01%; prednisolone, 0.07%; cortisol, 0.23%; cortisone, < 0.01%; progesterone, 2.65%; testosterone 0.64% and estradiol 17β, < 0.01% (Santymire and Armstrong, 2010). The EIA was validated biochemically by demonstrating (i) parallelism between binding inhibition curves of sera and the corticosterone standard (*r* = 0.991) and (ii) significant recovery (>90%) of exogenous corticosterone (1.95–1000 pg/well) added to serum (*y* = 0.9947*x* − 0.3078; *R*^2^ = 1.0; *P* < 0.001). Assay sensitivity was 3.9 pg/well and intra- and inter-assay coefficients of variation were <10%.

Cortisol polyclonal antiserum (R4866; Munro, University of California-Davis, CA) and HRP were used at dilutions of 1:8500 and 1:20000, respectively (Loeding *et al*., 2011). Cross-reactivities of cortisol R4866 antibody are reported as cortisol 100%, prednisone 6.3%, corticosterone 0.7%, 21-deoxycorticosterone 0.5%, progesterone 0.2%, pregnenolone 0.1%, androstenedione 0.1%, dehydroisoandrosterone-3-sulfate 0.1%, estradiol-17β 0.1%, estriol 0.1%, cholesterol 0.1%, prednisolone 9.9%, cortisone 5.0%, deoxycorticosterone 0.3%, 11-deoxycortisol 0.2%, 17α-hydroxyprogesterone 0.2%, 17α-hydroxypregnenolone 0.1%, testosterone 0.1%, dehydroepiandrosterone 0.1%, aldosterone 0.1%, estrone 0.1% and spironolactone 0.1% (Young *et al*., 2004). The EIA was biochemically validated by demonstrating (i) parallelism between the binding inhibition curves of sera and the cortisol standard (*r* = 0.988) and (ii) significant recovery (>90%) of exogenous cortisol added to serum (*y* = 0.9619*x* + 1.9893; *R*^2^ = 1.0; *P* < 0.001). Assay sensitivity was 3.9 pg/well and intra- and inter-assay coefficients of variation were <10%.

### Data analyses

For the biochemical validation, Pearson’s product moment correlation was used to compare the relationship of the parallelism between the corticosterone or cortisol standards and serially diluted serum pool. For the percent recovery, we graphed a scatterplot of the observed over expected values of samples spiked with the corticosterone or cortisol standards and did a linear regression to get the equation of the best fit line and *R*^2^ value for each species. For the physiological validation, a repeated measures analysis of variance (RM ANOVA) was used to compare an individual’s response to both the saline and ACTH over time. Within treatment, the effect of an individual’s age and sex on the response was determined using a RM ANOVA. All statistical analyses were performed using Microsoft Excel (Microsoft Corp., Redmond, WA, USA) and SigmaPlot (2008, v 11; Systat Software, Inc., San Jose, CA, USA). Values are presented as mean ± SEM. For all analyses, *P* < 0.05 was considered significant.

## Results

### Corticosterone analysis

**Figure 2:**
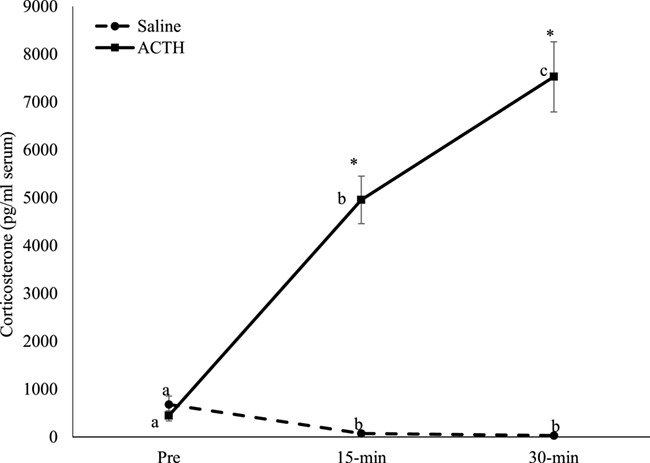
Mean (±SEM) corticosterone concentrations from 14 sea otters (5 males; 9 females) after a saline (control) and ACTH injection. Asterisks indicate differences (*P* < 0.001) between treatments at the different time periods. Different superscripts indicate differences (*P* < 0.01) among time periods within the treatment groups

In both the saline and ACTH treatments, sex (saline, *F*_1,24_ = 0.656; *P* = 0.434; ACTH, *F*_1,24_ = 3.708; *P* = 0.078) and age (saline, *F*_3,20_ = −0.972; *P* = 0.444; ACTH, *F*_3,20_ = 1.683; *P* = 0.223) category did not affect the change of corticosterone over time; therefore, all individuals were combined for further analysis. Overall, the trials (saline versus ACTH) produced different (*F*_1,26_ = 104.836; *P* < 0.001) responses. Specifically, corticosterone concentrations at pre-injection were similar (*q* = 0.647; *P* = 0.651), but the ACTH corticosterone response was higher than saline at 15 min (*q* = 13.303; *P* < 0.001) and 30 min (*q* = 20.429; *P* < 0.001) post-injection (Fig. 2). Within the control trials, corticosterone varied (*X*^2^ = 24.133, df = 2, *P* < 0.001) across time. Specifically, corticosterone was higher at the pre-treatment (mean, 681.03 ± 169.73 pg/ml) compared to the 15-min (mean, 71.58 ± 31.63 pg/ml; *q* = 6.236; *P* < 0.05) and 30-min (mean, 30.01 ± 14.15 pg/ml; *q* = 6.013; *P* < 0.05) post injection, but 15- and 30-min were similar (*q* = 2.268; *P* > 0.05; Table 1; Fig. 2). Following ACTH administration, serum corticosterone did vary (*X*^2^ = 26.143, df = 2, *P* < 0.001) over time. Specifically, corticosterone increase from pre-treatment (mean, 443.50 ± 106.46 pg/ml) to 15-min post-injection (mean, 4953.06 ± 663.43 pg/ml; *q* = 5.669; *P* < 0.05) and 30-min post-injection (*P* < 0.05; mean, 7526.39 ± 732.03 pg/ml; *q* = 7.216; *P* < 0.05) and 30-min was higher (*q* = 4.536; *P* < 0.05) than 15-min (Table 1; Fig. 2). From pre- to 15-min, there was a mean 1039.48 ± 578.41-fold (range, 2.9- to 6745.7-fold) increase. From 15 to 30 min post-ACTH injection, the mean fold increase was 1.6 ± 0.1 (range, 1.0–2.7-fold). Thirteen of the 14 sea otters (except 696–15) had corticosterone values that were still increasing at 30 min post-ACTH (*y* = 3541.4*x* − 2775.2; *R*^2^ = 0.976; except 696–15).

**Table 1:** Corticosterone (pg/ml serum) results for control (saline) and ACTH injections

	**Control injection**			**ACTH injection**		
**Sea otter**	**Pre**	**15-min post**	**30-min post**	**Pre**	**15-min post**	**30-min post**
479	648.48	0	0	674.58	1989.46	5322.80
567	2155.02	0	0	497.72	3887.48	6546.56
595	896.42	0	0	497.72	3887.48	6546.56
623	473.54	0	0	523.74	7411.86	15378.80
649	1233.50	178.24	0	1185.10	8316.74	8395.16
654	82.52	0	0	0	6745.70	8568.18
657	459.22	371.78	86.38	75.44	3779.28	7194.20
671	535.32	0	0	0	3029.14	6264.74
673	747.06	0	0	1268.48	4154.74	7103.90
684	77.60	28.6	26.24	355.40	5206.50	8615.16
685	480.36	95.70	85.84	262.18	6900.12	9109.62
687	66.44	63.50	45.20	451.38	5523.12	7703.28
696	1678.92	264.26	177.72	416.64	5370.48	5003.10
5059	0	0	0	0.68	3140.72	3617.38

**Figure 3:**
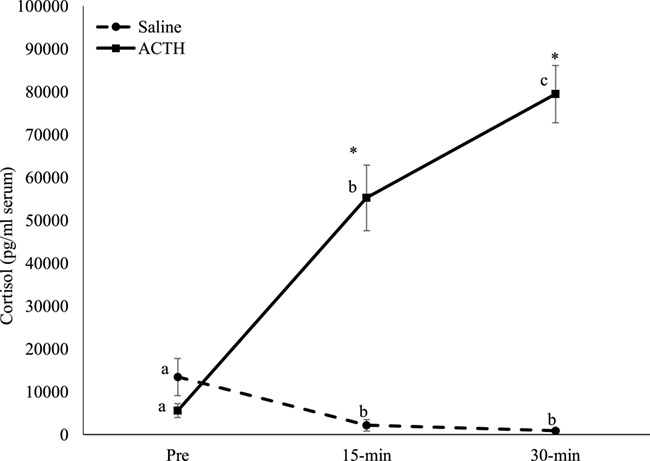
Mean (±SEM) cortisol concentrations from 14 sea otters (5 males; 9 females) after a saline (control) and ACTH injections. Asterisks indicate differences (*P* < 0.001) between treatments at the different time periods. Different superscripts indicate differences (*P* < 0.01) among time periods within the treatment groups

**Table 2:** Cortisol (pg/ml serum) results for control (saline) and ACTH injections

	**Control injection**			**ACTH injection**		
**Sea otter**	**Pre**	**15-min post**	**30-min post**	**Pre**	**15-min post**	**30-min post**
479	546.20	6.96	0	1030.42	3418.80	59686.40
567	9277.20	436.36	50.72	1656.56	43031.60	50957.60
595	28723.20	1382.92	835.34	19881.60	64177.60	85074.40
623	3040.84	1030.30	760.56	4055.94	42636.00	61689.60
649	33939.00	1517.06	902.30	4432.54	42152.80	66478.40
654	11490.60	3790.80	973.98	2195.98	63488.80	76574.40
657	37731.80	19040.00	7233.80	2679.26	40903.80	71820.00
671	288.60	86.42	6.80	441.78	80204.00	92520.00
673	1971.52	190.12	43.44	3428.08	83676.00	110575.20
684	577.40	291.56	217.48	10658.52	61268.80	76103.20
685	13889.88	1105.54	642.04	1909.52	117739.20	149685.60
687	539.76	397.70	253.88	12241.08	63788.80	74446.40
696	46167.30	1383.70	909.42	14135.22	52822.40	67314.40
5059	125.58	0	0	250.42	14369.40	69615.20

### Cortisol analysis

Within the control and ACTH groups, sex (saline, *F*_1,24_ = 0.072; *P* = 0.794; ACTH, *F*_1,24_ = 0.0004; *P* = 0.984) and age (saline, *F*_3,20_ = −0.485; *P* = 0.700; ACTH, *F*_3,20_ = 2.060; *P* = 0.169) category did not affect cortisol over time; therefore, all individuals were combined for further analysis. Overall, the trials (saline versus ACTH) produced different (*F*_1,26_ = 66.814; *P* < 0.001) responses. Specifically, serum cortisol concentrations at pre-injection were similar (*q* = 1.720; *P* = 0.234), but the ACTH cortisol response was higher than saline at 15-min (*q* = 11.693; *P* < 0.001) and 30-min (*q* = 17.307; *P* < 0.001) post-injection (Fig. 3). Within the control trials, cortisol varied (*X*^2^ = 27.527, df = 2, *P* < 0.001) across time. Specifically, cortisol was higher at the pre-treatment (mean, 13450.63 ± 4337.61 pg/ml) compared to the 15-min (mean, 2189.96 ± 1323.16 pg/ml; *q* = 5.480; P < 0.05) and 30-min post injection (mean, 916.41 ± 497.05 pg/ml; *q* = 7.350; *P* < 0.05; Table 2; Fig. 3). Additionally, 15-min was higher (*q* = 4.914; *P* < 0.05) than 30-min post-injection of saline. Following ACTH administration, serum cortisol did vary (*X*^2^ = 28.00, df = 2, *P* < 0.001) over time. Serum cortisol increased from pre-treatment (mean, 5642.64 ± 1624.57 pg/ml) to 15-min post-injection (mean, 55262.71 ± 7646.62 pg/ml; *q* = 5.292; *P* < 0.05) and 30-min post-injection (mean, 79467.20 ± 6686.51 pg/ml; *q* = 7.483; *P* < 0.05) and 30-min was higher (*q* = 5.292; *P* < 0.05) than 15-min (Table 2; Fig. 3). From pre- to 15-min there was a mean 31.2 ± 12.6-fold (range, 3.2- to 181.5-fold) increase. Within the 15- to 30-min post-ACTH interval the mean fold increase was 2.7 ± 1.2 (range, 1.2–17.5-fold). All 14 sea otters had cortisol values that were still increasing at 30-min post-ACTH (*y* = 36 912*x* − 27 034; *R*^2^ = 0.962).

### Glucocorticoid comparison

**Figure 4:**
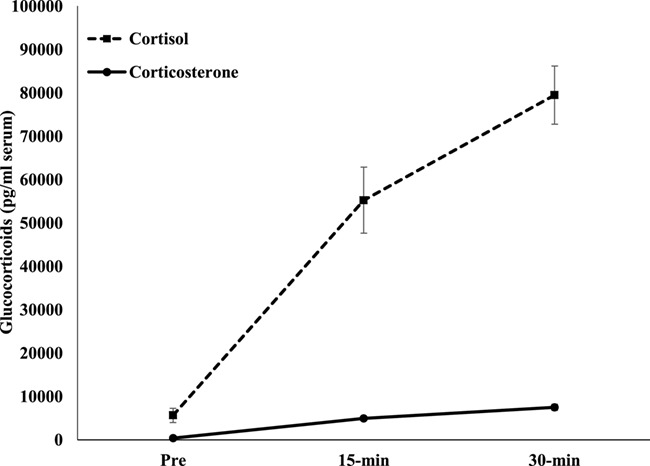
Mean (±SEM) glucocorticoid concentrations from 14 sea otters (5 males; 9 females) after ACTH injections

There were higher quantities of cortisol than corticosterone based on dilution rates and the amount of hormone each EIA was able to measure (both have a sensitivity of 3.9 pg/well; Fig. 4). Samples had to be diluted from neat to 1:120 for cortisol EIA and from neat to 1:9 for corticosterone EIA even after the ACTH. Both cortisol and corticosterone demonstrated an unanticipated change within the control treatment. While both hormones were detected at pre-injection, levels had decreased at 15 min post-saline injection and had returned to pre-injection concentrations at 30 min.

## Discussion

This was the first controlled study in which the blood-based response to an ACTH challenge is used to compare the level of response in the form of cortisol and corticosterone in the sea otter. Our study demonstrates that cortisol is the predominant glucocorticoid when the HPA axis is activated. Sex and age did not affect how the individual responded to the ACTH or saline injection. Interestingly, the saline injection not only confirmed the effects of the ACTH on glucocorticoid release from the adrenal glands but also provided information on how long it takes the sea otter’s glucocorticoid levels to return to baseline after capture and sedation.

Determining the predominant glucocorticoid for a species is important for monitoring how an individual is coping with the changing environment (Palme, 2005). However, because cortisol and corticosterone share similar synthesis pathways and enzymes, a species that has cortisol as the predominant glucocorticoid may still produce corticosterone and vice versa (Koren *et al*., 2012). For instance, Koren *et al*. (2012) found that in cortisol-dominant species, 70% (9 of 13 species) had a positive association between cortisol and corticosterone. Here, we found that corticosterone and cortisol responded similarly to the ACTH; however, corticosterone increased at a higher magnitude. In the cortisol-dominant female yellow-pine chipmunks (*Tamias amoenus*), cortisol and corticosterone mirrored each other suggesting both have a role during HPA axis activation (Kenagy and Place, 2000). Conversely, the two GCs had opposing fluctuations over different seasons in the golden-mantled ground squirrel (*Spermophilus saturatus*; Boswell *et al*., 1994). Each GC may vary in functionality, including its role in different age classes (Palme, 2005), circannual cycles (Boswell *et al*., 1994) and metabolic effects (Devenport *et al*., 1989). Their function may be related to binding affinity to corticosteroid-binding globulin (CBG) for blood transportation and differing brain receptors for anabolism versus catabolism (Devneport *et al*., 1989; Kenagy and Place, 2000). Determining GCs varies functionality would require measuring free and CBG-bound concentrations, along with GC receptors and their locations (Place and Kenagy, 2000).

The stimulation of the HPA axis can be a psychological and/or a physiological trigger. How an individual responds to a stressor is also dependent on an individual’s experience, genetics, health and environment both social and physical (Romero, 2004; Touma and Palme, 2005). The reactivity of both GCs to the stimulation by ACTH may also vary (Venkataseshu and Estergreen, 1970). In Rusa deer (Cervus *Rusa timorensis*), cortisol and corticosterone followed a similar pattern post-ACTH injection; however, cortisol concentrations were higher and had a higher response (~25-fold increase) compared to corticosterone (~21-fold increase; Stelmasiak and Outch, 1985). In the tuco-tucos (*Ctenomys talarum*), cortisol was found to be the more responsive GC to an acute stressor (Vera *et al*., 2011). Here, we found that both cortisol and corticosterone increased 15 min post-ACTH administration and continued to rise until 30 min post-injection. The study design was such that we elected not to maintain chemical immobilization beyond 30 min; therefore, it is unclear how long or elevated these levels would be. The most dramatic increase in serum levels occurred within the first 15 min (3- to 182-fold higher than pre-injection in cortisol; 3- to 4618-fold higher than pre-injection values in corticosterone) compared to the 30-min post-injection (1- to 17-fold higher in cortisol from the 15-min time period; 1- to 3-fold higher in corticosterone from the 15-min time period); therefore, it is believed that the rate of increase would continue to decline until reaching a maximum level at some point beyond 30 min. A similar study was conducted in captive coyotes (*Canis latrans*) where the authors were able to keep the coyotes under anaesthesia for 90 min during an ACTH challenge. They found that male plasma cortisol plateaued at 60 through 90 min post-ACTH. In females, plasma cortisol peaked at 60 min post-ACTH (Stevenson *et al*., 2018). In the Stellar sea lion (*Eumetopias jubatus*), individuals remained under anaesthesia for 3 to 4 h during an ACTH challenge and plasma cortisol peaked at 195 min, but only increased 1-fold higher after 60 min (Mashburn and Atkinson, 2004). In a similar study, Mashburn and Atkinson (2008) found serum cortisol peaking at 90 min post-ACTH in Stellar sea lions.

Although we are the first to use an ACTH challenge to measure blood GC responses, Wasser *et al*. (2000) conducted an ACTH challenge on an Alaskan sea otter to develop techniques for faecal glucocorticoid metabolite (FGM) analysis. The authors compared cortisol and corticosterone hormone assays and determined that cortisol was the predominant FGM (Wasser *et al*., 2000). ACTH challenge testing has been used in another mustelid, the black-footed ferret (*Mustela nigripes*; Young *et al*., 2001, 2004). Although the authors did not measure blood GCs, they did compare cortisol and corticosterone via FGM analysis and found that either GCs would be an effective technique to monitor adrenocortical activity (Young *et al*., 2001).

In addition to physiological validations, like ACTH challenges, perceived stress events, such as capture, restraint, immobilization and transportation, also can be used to validate GC analysis (Touma and Palme, 2005). For example, veterinary examination was used to validate FGM analysis in captive northern river otters (*Lontra canadensis*; Rothschild *et al*., 2008). Larson *et al*. (2009) found that capturing wild sea otters using a Wilson trap resulted in significantly higher serum corticosterone levels compared to capture with a tangle net. The time between capture and immobilization was shorter with the Wilson trap (62 vs. 79 min) and that may have affected GC production (Larson *et al*., 2009). Here, we observed measurable levels of both corticosterone and cortisol at 10 min post handling for the saline injection control. The significant drop in GC levels within 15 min (i.e. 25 min post handling) may have implications for use in field and laboratory settings. Data presented suggests that chemical sedation with fentanyl and midazolam coupled with the 8- to 10-min induction period within a dark, quiet induction box allows serum glucocorticoids to drop to non-detectable levels within a relatively short time period. Similarly, captive male and female coyotes had plasma cortisol that was elevated through a 60-min post-saline injection even though this group was the control group for the ACTH challenge (Stevenson *et al*., 2018). In another captive coyote study, FGMs of control individuals had similar elevated peaks, particularly the females, compared with ACTH-injected individuals most likely due to handling stress (Schell *et al*., 2013). Elevated FGMs post-saline injection was also observed in the black-footed ferret (Young *et al*., 2004).

Interestingly, Larson *et al*. (2009) found that corticosterone was the most responsive GC in wild sea otters when investigating the HPA activation immediately post-capture. Here we found that cortisol was the predominant blood GC. This difference could be a result of type of hormonal assays used (Larson and colleagues used radio immunoassays). Additionally, Larson *et al*. (2009) reported similar cortisol results 33 ng/ml in California males and 49 ng/ml in females (here, < 1 to 20 ng/ml in pre-injection samples). However, they reported corticosterone concentrations between 25 and 39 ng/ml in males and females, respectively. We observed corticosterone values < 1 to 2 ng/ml. This difference may also be explained by capture methods and timing because our sea otters were captive compared to their wild-caught individuals. As mentioned above, we even observed lower cortisol and corticosterone plasma values 30 min post-saline injection with the saline injection. Larson *et al*. (2009) also reported a sex effect with females (from Alaska and Washington) producing higher GC values than males. We did not observe a sex nor an age effect on blood GC responses to the ACTH nor saline. The difference in GC between the sexes might be driven by physiological factors, such as seasonality, reproductive state, and behavioural factors, including social pressures and territoriality (Touma and Palme, 2005). Typically, juveniles have higher GC values than adults most likely because they are growing and have a faster metabolic rate (Millspaugh and Washburn, 2004).

This study successfully answered the question regarding predominant glucocorticoid in the sea otter by measuring response to ACTH challenge over a relatively short time period. By itself, this information has little application beyond furthering the understanding of basic sea otter physiology. With this information, however, it becomes possible to consider the use of minimally to non-invasive methods to evaluate stress in free ranging sea otter populations. It is highly likely that sea otters, like other mammals, have some degree of diurnal cycling of GCs, now known to be cortisol in the sea otter. Therefore, single, random sampling of traditional body fluids, such as blood, urine and saliva, or faeces may not provide an interpretable measurement of GC production and responses. If the methodology for determining cortisol levels in more durable tissue, such as hair, can be validated, numerous opportunities may be recognized. Cortisol levels in structures such as hair tend not to be subject to the effects of the diurnal variability and tend to represent more stable and interpretable data associated with glucocorticoid levels evened out over time (Gow *et al.*, 2010; MacBeth *et al.*, 2010; Russell *et al.*, 2012; Sheriff *et al*., 2011). These data are far more useful in field settings in which animals are typically only handled once. Sea otters also present a somewhat unique opportunity to look into their historical record. Sea otter fur was prized for its density and luxurious texture. As a result, pelts were historically used for a variety of clothing and ornamental purposes. Many of these items, including some from prehistoric times, remain. If cortisol reliably persists over time in fur, it may be possible to go back to evaluate stress in historic populations (Bechshoft *et al.*, 2012). This study is but the first of several which may provide the opportunity to better understand the role of stress hormones in sea otter populations.
